# Active Surveillance of Patients Colonized with CRE: A Single-Center Study Based on a Combined Molecular/Culture Protocol

**DOI:** 10.3390/antibiotics13111053

**Published:** 2024-11-06

**Authors:** Beatrice Silvia Orena, Maria Francesca Liporace, Antonio Teri, Daniela Girelli, Federica Salari, Michela Mutti, Gabriele Giordano, Claudia Alteri, Flaminia Gentiloni Silverj, Caterina Matinato, Annapaola Callegaro, Lisa Cariani

**Affiliations:** 1 Clinical Microbiology and Virology Unit, Fondazione IRCCS Ca’ Granda Ospedale Maggiore Policlinico, 20122 Milan, Italymariafrancesca.liporace@policlinico.mi.it (M.F.L.); gabriele.giordano@policlinico.mi.it (G.G.); claudia.alteri@unimi.it (C.A.);; 2 Residency in Microbiology and Virology School, University of Milan, 20122 Milan, Italy; 3 Department of Oncology and Hemato-Oncology, University of Milan, 20122 Milan, Italy; 4 Direzione Medica di Presidio, Fondazione IRCCS Ca’ Granda Ospedale Maggiore Policlinico, 20122 Milan, Italy

**Keywords:** active surveillance, antimicrobial resistance, carbapenem-resistant *Enterobacteriaceae*, culture-base method, molecular screening

## Abstract

**Background/Objectives**: Carbapenem-resistant *Enterobacteriaceae* (CRE) are types of bacteria that need urgent attention globally. Active surveillance programs at hospitals are essential for the early identification of CRE carriers and the timely adoption of infection control measures. We aimed to analyze the epidemiology of CRE identified by multiplex RT-PCR in rectal swabs of patients upon admission to high-risk wards and to compare data obtained from both molecular and culture CRE screening. **Methods**: A total of 2861 rectal swabs, prospectively collected within 12–24 h of admission, underwent molecular screening for identification of *K. pneumoniae* carbapenemase (KPC), New Delhi metallo-β-lactamase (NDM), Verona integron-mediated metallo-β-lactamase (VIM), imipenemase (IMP), and OXA-48 (Allplex^TM^ Entero-DR Assay). Only samples that tested positive or invalid underwent culture testing (Agar MacConkey and CHROMID^®^ CARBA plates, bioMérieux, Craponne, France). **Results**: A total of 118 out of 2861 (about 4%) were positive for at least one carbapenem-resistant gene by a molecular approach (MA), with KPC, NDM, and VIM having the highest prevalence. Culture testing confirmed the presence of carbapenemase in 89 samples (75.4%), showing a disagreement rate of about 25% between the two methods, which, unfortunately, rises up to 60% for VIM. The dominant bacterial species were *K. pneumoniae* and *E. coli* (MALDI-TOF mass spectrometry). **Conclusions**: Our data underlined the need for the molecular screening of CRE carriers in order to implement active surveillance protocol in critical care settings and to improve infection control measures.

## 1. Introduction

Antimicrobial resistance (AMR) is a major global public health threat, with an estimated 4.95 million deaths in 2019. Carbapenem-resistant *Enterobacteriaceae* (CRE) is one of the top three critical multi-drug-resistant pathogens on the priority list of the World Health Organization (WHO) [1]. The United States Center for Disease Control and Prevention (CDC) defined CRE as *Enterobacteriaceae* with in vitro resistance to at least one carbapenem [2]. Carbapenems are β-lactam antibiotics with broad-spectrum activity that work by inhibiting penicillin-binding proteins (PBPs) [3]. For a long time, carbapenems were considered an important therapeutic option to treat challenging infections; therefore, resistance to this antibiotic class has significantly narrowed antibiotic choices in critically ill patients, with associated increases in morbidity and mortality [3,4,5].

The prevalence of colonization and infection with CRE has been increasing globally, especially in healthcare settings [4,5]. More than 99% of *Enterobacteriaceae* were susceptible to carbapenems before 2001, while in the years 2013, 2014, and 2015, it was reported that 1.3/10,000 patients in Europe and 4/10,000 patients in China were infected with CRE, with an increase of more than 60% in 2020 [6,7]. Furthermore, several epidemiological studies have highlighted that *Klebsiella pneumoniae* and *Escherichia coli* count for approximately 90% of all CRE, and they are distributed worldwide [6,8]. 

In recent years, the Italian AMR situation has been repeatedly defined as one of the worst among European countries by the European Centre for Disease Prevention and Control (ECDC). The number of AMR deaths in Italy is higher than those from chronic respiratory diseases, digestive diseases, respiratory infections, and tuberculosis, with 8800 deaths attributable to AMR and 35,800 deaths associated with AMR in 2019 [9,10]. *Escherichia coli* and *Klebsiella pneumoniae* are among the top five pathogens to be aware of in Italy based on the number of deaths associated with AMR; *Escherichia coli* and *Klebsiella pneumoniae* are responsible for 11,300 and 4200 deaths, respectively [10]. 

Since *Enterobacteriaceae* are common commensals of gut microbiota, and because carbapenem-resistant microorganisms can be considered long-term persistent colonizers, intestinal colonization of CRE in hospitalized patients functions as a reservoir for disseminating these pathogens in hospital settings [5,10,11]. CRE rectal screening based on culture techniques still represents the gold standard for its high specificity and reproducibility rates, even if it requires a long turn-around time (TAT) [12]. In order to ensure timely implementation of infection control measures, numerous evidence supports the use of active surveillance programs for high-risk patients. Indeed, the detection of carbapenemase genes directly from rectal swabs leads to a TAT reduction (from 18–24 h to 4 h), and it can be considered a key aspect in the management of colonized patients (rapid patient isolation and activation of contact precautions) [13,14,15,16]. Furthermore, the molecular detection of CRE allows the detection of bacteria with low carbapenem MICs (resistance genes expressed at low levels), which may not grow on selective culture media [17,18]. Lastly, the ECDC reports that active rectal screening at the time of admission to a hospital (or a specific ward) and periodic screening during both hospitalization and an outbreak can effectively limit and prevent the spread of CRE [12]. 

In this context, our hospital has introduced active surveillance programs for all patients admitted to high-risk wards starting from 1 January 2024. The aims of this study were to analyze the epidemiology of CRE identified by MA in rectal swabs of patients at hospitalization and to compare the active surveillance data obtained from CRE screening by the molecular approach (MA) and the conventional culture-based approach (CA). 

## 2. Results

### 2.1. Sample Collection and CRE-Positivity Rate by Molecular Test

A total of 2861 rectal swabs were prospectively collected from as many patients as possible who were admitted to high-risk wards from 1 January to 30 June 2024. A total of 141 rectal swabs were collected from the hematology unit, 296 from the intensive care unit, 2010 from the medicine unit, and 414 from the surgery unit. The distributions of rectal swabs relative to the units and periods are reported in Table 1. 

Out of 2861 rectal swabs, 118 (4.1%) were positive for at least one CRE gene by MA, 2521 (88.1%) tested negative, and 222 (7.8%) tested invalid. The prevalence of CRE did not differ significantly against months (*p*-value = 0.23), nor did it when single wards were considered (Table 1). The CRE prevalence was highest in the medicine ward (4.7%) with respect to the others (intensive care: 3.7%, hematology: 3.5%, and surgery: 1.6%; *p* = 0.04).

**Figure 1:**
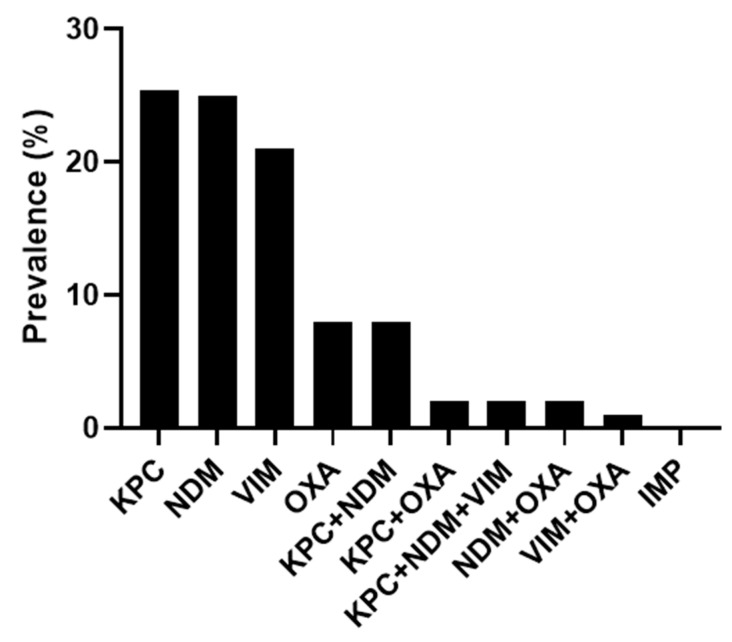
Distribution of CRE genes detected by MA.

Of the 118 tested positive samples, 95 (80.5%) carried one CRE gene. In particular, KPC and NDM were found alone in 30 samples each (25.4%), while VIM and OXA-48 were found alone in 25 (21.3%) and 10 (8.5%) samples, respectively. The remaining 23 samples (19.5%) showed more than one CRE gene. Most of them carried KPC+NDM (n = 10), followed by KPC+OXA-48 (n = 3), KPC+NDM+VIM (n = 3), NDM+VIM (n = 3), NDM+OXA-48 (n = 3), and VIM+OXA-48 (n = 1). The IMP gene was not detected in any rectal swabs (Figure 1).

### 2.2. Distribution of CRE Against Bacteria Species

**Figure 2:**
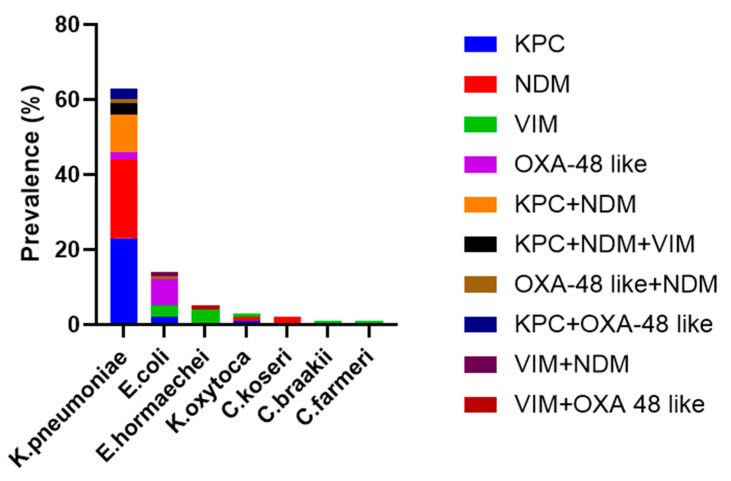
Prevalence of bacterial species identified by CA against antimicrobial-resistant genes.

Looking at the 89 CA isolates, the two most prevalent were *K. pneumoniae* (69.7%, n = 62) and *E. coli* (15.7%, n = 14), followed by *Enterobacter hormaechei* (5/6, n = 5) (Figure 2). As expected, KPC and NDM, alone or when in combination, were prevalently found in *K. pneumoniae* (97.5 and 87.5%, respectively). OXA-48 and VIM were prevalently detected in *E. coli* and *E. hormaechei* (60% each). Moreover, *K. pneumonaie* was the main bacterial species that expressed a combination of a more carbapenem-resistant mechanism (KPC+NDM, KPC+NDM+VIM, and KPC+OXA-48).

### 2.3. Concordance Rate with Culture Method

When CRE-positive samples according to MA were tested according to CA, the gold standard method confirmed positivity to carbapenemase in 89 of them (75.4%). The results were thus discordant (+/−) for 29 samples. To confirm this point, we repeated CA in three follow-up swabs (as required by the protocol in use) for the 29 discordant samples, and the resistance mechanism detected by the MA was not confirmed by CA for any of them.

**Figure 3:**
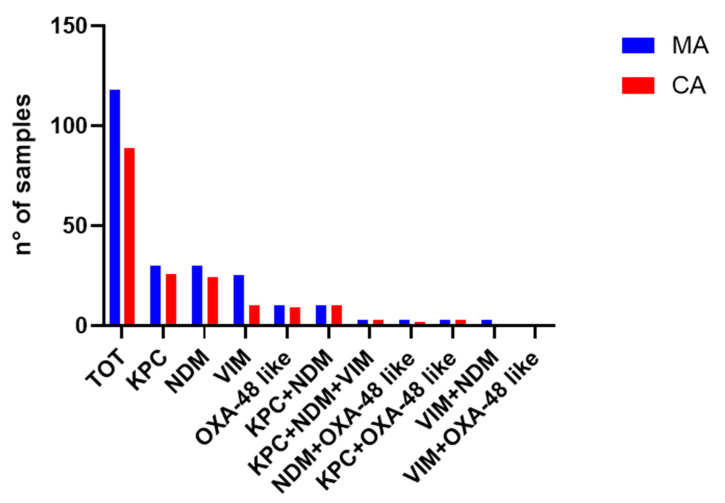
Concordance between MA and CA.

In samples carrying one CRE gene, the concordance rate (+/+) between MA and CA was 86.7% for KPC (26/30), 80% for NDM (24/30), 40% for VIM (10/25), and 90% for OXA-48 (9/10) (Figure 2). The copresence of enzymes from different families was always correctly detected for KPC+NDM (100%, 10/10), KPC+NDM+VIM (100%, 3/3), KPC+OXA-48 (100%, 3/3), and VIM+OXA-48 (1/1), with the exception of NDM+OXA-48 and VIM+NDM combinations, which were identified by both MA and CA in cases 1 and 2, respectively (Figure 3).

Of the 222 rectal swabs tested invalid by MA, 148 (66.7%) were negative according to CA, and 74 (33.3%) were defined as unsuitable samples because growth was absent on bacterial media.

Overall, the concordance rates obtained between the two methods could be higher if it were not for the 60% VIM disagreement rate (Figure 3).

## 3. Discussion

The rapid spreading of carbapenemase-producing *Enterobacterales* is reported worldwide in clinical settings. Because intestinal colonization by CRE can persist for long periods, the condition of carriers is considered one of the most important risk factors for infection. CRE infections are associated with fast interpatient transmission and high mortality rates; thus, early identification of CRE carriers is globally considered a key point for the containment of these organisms. This is why molecular detection of carbapenemase genes from rectal swabs represents a useful tool for patients’ management and timely application of infection control protocol [13,19,20].

Our work has allowed us to outline the epidemiology of CRE carriers in our hospital. In particular, the overall CRE prevalence has been assessed at around 4%, mostly due to KPC and NDM resistance genes carried by *K. pneumoniae* and *E. coli*. The data obtained are in line with Italian epidemiology, which indicates that the prevalence range of CRE colonization in hospitalized patients is 3–7%. Moreover, as obtained in our study, the literature reports *K. pneumoniae* as the most common CRE species, which is followed by in increasing order of *E. coli*, *Klebsiella oxytoca* and *Enterobacter cloacae* [19,21,22,23]. 

Outside Europe, Wangchinda and colleagues reported a prevalence of 12.6% for newly detected CRE colonization among patients upon admission to general medicine wards in a Thai university hospital in a study period between 2018 and 2021 [5]. This prevalence is much higher than that reported in our manuscript and in Italian epidemiology [19,21,22,23]; nonetheless, there is a longer study period with fewer analyzed samples. In line with our findings, they obtained a similar prevalence for the most common CRE species: more than 75% of *K. pneumonia* and almost 20% of *E. coli* [5]. In a similar way, Gomides and colleagues analyzed 3154 rectal swabs collected from patients admitted to an adult intensive care unit in a southeastern Brazilian hospital from 2014 to 2018. They reported a CRE prevalence of almost 11% and *K. pneumoniae* as the most common species (about 83%) [24]. Overall, these comparisons highlight the importance of each study, which contributes its own reality to the global monitoring of CRE epidemiology.

Our work also highlights that MA positivity has been confirmed in over 75% of samples, according to CA. This concordance reached 86.7%, excluding the VIM gene, which accounts for more than 50% of discordant results. These data are consistent with the literature published thus far [19,25], which highlights that molecular detection of CRE offers equal or higher sensitivity relative to the culture-based method; LODs are variable from method to method and are usually gene-dependent. However, a risk of MA to take into account is the possibility of detecting dead bacteria, so the presence of viable CRE may be overestimated, leading to a negative result in culture tests but a positive result in molecular tests [26]. In addition, positive results with MA but negative results with CA may happen if the patient is on antibiotic therapy; the bacteria may carry a modified sequence of the target gene, which is either not expressed or is expressed at low levels [20]. Moreover, the detection of CRE directly from fecal swabs using chromogenic media depends on several factors, such as the chromogenic media brand, species, resistance mechanism, and type of inoculum (with or without a pre-enrichment step) [12]. All these hypotheses can explain the discordant results obtained in our study, even if further investigations are needed to define the source of this gene by using a metagenomic approach as a reference without the limitation of known targets, as is present for PCR-based assays. CRE is not the only threat in hospital settings. Among carbapenem-resistant gram-negative bacteria (CRGNB), it has been determined that carbapenem-resistant *Acinetobacter baumannii* (CRAB) and *Pseudomonas aerginosa* (CRPA) are leading causes of health-care associated infections [27,28] that need to be detected as rapidly as CRE. We present preliminary data on an RUO (Research Use Only) kit (Allplex^TM^ Entero-DR Plus Assay kit, Seegene, Seoul, Republic of Korea) we tested (an internal validation according to the laboratory’s quality criteria) in order to quickly implement the molecular screening of high-risk patients. This multiplex RT-PCR assay is able to simultaneously detect *A. baumanni* and *P. aeruginosa*. We enrolled 21 patients, and three swabs were collected for each one (inguinal, pharyngeal, and rectal swabs). These patients were randomly selected from those who were subjected to routine culture tests for *A. baumanii*. Even if the test was conducted with a RUO kit, we decided, in agreement with the medical director department, to promptly notify the involved wards in case a sample tested positive. From the molecular screening, six swabs (three inguinal, two rectal, and one pharyngeal swab) tested positive for CRAB, allowing for the timely adoption of preventative functional isolation methods with the application of specific contact precautions. All samples, including those that tested negative and positive, were confirmed by routine culture testing (based on the inoculation of 100 µL of medium-transported swabs from Agar MacConkey). The resistance mechanism was determined using the immunochromatographic RESIST ACINETO (CORIS, BioConcept, Gembloux, Belgium), which is able to detect the carbapenemases OXA-23, OXA-40/58, and NDM. All identified *A. baumannii* were OXA-23 producers. These data demonstrate the easy adaptability of molecular assays on different targets, depending on the diagnostic need. Our study has some limitations. The first limitation of our work is that rectal swabs found negative by MA have not been tested by CA, preventing us from defining the specificity of this molecular assay. Second, the prevalence of CRE could be underestimated due to the predefined targets of molecular screening. Third, we focused our attention only on patients at high risk of infection, with a possible overestimation of CRE colonization. Last, as is frequently done in antibiotic resistance surveillance studies, we developed our study only on microbiological data routinely collected by the laboratory without considering patient risk factors for CRE acquisition.

## 4. Materials and Methods

**Figure 4:**
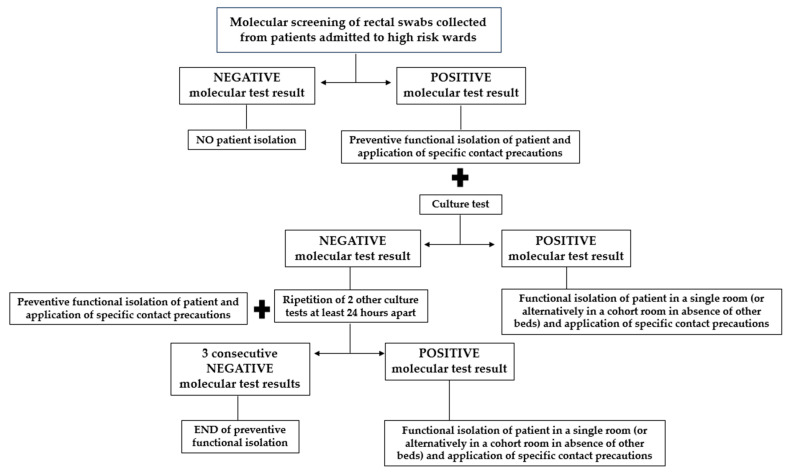
Hospital operating procedure on active surveillance for molecular CRE detection.

From 1 January 2024, Fondazione IRCCS Cà Granda Ospedale Maggiore Policlinico has introduced an active surveillance protocol for all patients admitted to selected high-risk wards, such as the hematology, intensive care, medicine, and surgery units within 12–24 h of admission. This protocol involves molecular screening of rectal swabs using gene amplification assays that detect CRE genes with a same-day response to clinicians. In the case of a negative molecular test result, a patient does not require isolation; in the case of a positive molecular test result, a patient is placed in preventive functional isolation with the application of specific contact precautions, pending a culture test result. In the case of a negative culture test result, it is necessary to maintain patient isolation until three consecutive negative culture tests (repeated at least 24 h apart) are obtained. However, in the case of a positive culture test result, a patient must be placed in a single room (or, alternatively, in a cohort room in the absence of other beds) in functional isolation with the application of specific contact precautions. The described operating procedure for active surveillance for molecular CRE detection is reported in Figure 4. 

**Figure 5:**
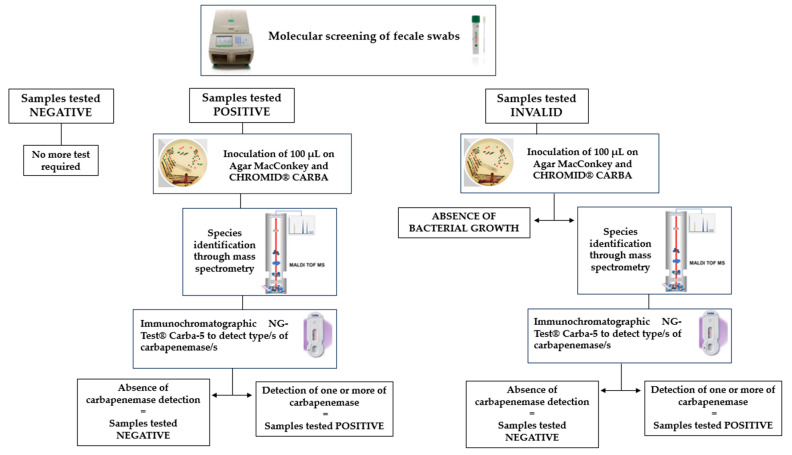
Description of all the stages in an active surveillance flowchart.

In this context, our prospective study was conducted from 1 January to 30 June 2024, with a total of 2861 rectal swabs collected from 2861 patients admitted to the aforementioned wards. All the samples were collected using Fecal Swab™ (COPAN Italia SpA, Brescia, Italy). Rectal swabs were transported to the microbiology and virology unit and processed on the same day. Specifically, all the collected samples underwent molecular screening (multiplex RT-PCR Allplex^TM^ Entero-DR Assay, Seegene), and only samples that tested positive or invalid underwent conventional cultured-based screening (Figure 5). A sample that tested invalid was defined as a sample with the possible presence of PCR inhibitors or with insufficient material. Figure 5 describes all the stages in an active surveillance flowchart.

### 4.1. Molecular Detection of Resistance Genes

Rectal swabs were processed using the Allplex^TM^ Entero-DR Assay kit (Seegene, Republic of Korea), which allows for the simultaneous identification of five carbapenemase resistance genes: *K. pneumoniae* carbapenemase (KPC, 96 variants), New Delhi metallo-β-lactamase (NDM, 18 variants), Verona integron-mediated metallo-β-lactamase (VIM, 70 variants), imipenemase (IMP, 56 variants), and OXA-48 like (20 variants). DNA extraction was carried out using STARMag 96 × 4 Universal Cartridge Kit (Seegene) on an automatic system Nimbus IVD (Seegene), with 300 µL of primary sample and 100 µL of DNA elution. Five microliters of DNA extract was mixed with 15 µL of PCR Mastermix, and RT-PCR was performed using a CFX96 system (Bio-Rad, Hercules, CA, USA). All procedures were performed according to the manufacturer′s instructions. The test results were interpreted automatically and presented using the Seegene Viewer software (ver 3.30.000). All the procedures were performed according to the manufacturer′s instructions using positive and negative controls provided by the kit in each assembly. The test results were interpreted automatically and presented using Seegene Viewer software (ver 3.30.000).

### 4.2. Conventional Culture-Based Method 

Microbiological diagnostics were performed in the respective routine microbiological laboratory following the current national standards and requirements.

All rectal swabs that tested positive or invalid with MA were subsequently analyzed with the conventional approach (CA) based on inoculation of 100 µL of a transport medium with fecal swabs on Agar MacConkey and CHROMID^®^ CARBA systems. The plates faced a 37 °C overnight incubation period, and the grown bacteria were identified by matrix-assisted laser ionization/desorption time-of-flight mass spectrometry (VITEK^®^ MS PRIME MALDI-TOF, bioMérieux, Craponne, France). To confirm the MA results, the production of carbapenemases was determined using the immunochromatographic NG-Test^®^ Carba-5 (NG Biotech, Guipry-Messac, France). This qualitative assay was able to detect the five most common carbapenemase families (KPC, OXA-48, VIM, IMP, and NDM), including a total of 76 variants. 

### 4.3. Statistical Evaluation and Comparisons 

The concordance rate was calculated as the proportion of concordant pairs over the sum of concordant and discordant pairs. In case of discordant results, patients were kept in isolation for up to three consecutive culture-negative rectal swabs. Categorical variables were analyzed using the chi-square test for trend. All statistical analyses were performed using SPSS for Windows software, version 29.0.1.0 (SPSS Inc., Chicago, IL, USA)

## 5. Conclusions

This prospective study clearly describes CRE epidemiology in our hospital, with a CRE prevalence of about 4%; this prevalence level has occurred mainly due to the presence of KPC and NDM as carbapenem-resistant genes and *K. pneumonia* as the dominant bacterial species. Notably, all our data (including data about *A. baumannii*) reinforce the indispensable nature of molecular assays for the rapid detection of multi-drug-resistant microorganisms. Fortunately, the commercial landscape of these assays offers a wide range of products that can be easily modeled on different gene targets. 

Moreover, our data report a concordance rate between MA and CA of 75.4%, highlighting that CRE molecular detection has a greater sensitivity than the culture-based method. Molecular screening offers a useful and fast method for the early detection of CRE genes among clinical isolates, making it possible to implement active surveillance. 

To conclude, we must not forget that active surveillance carried out by the laboratory represents a single, indispensable piece of the fight against antibiotic resistance. These data need to be integrated with hospital infection control practices routinely implemented in hospital settings.

## Figures and Tables

**Table 1:** Distribution of rectal swabs relative to the units, periods, and CRE-positivity rates.

Months	Number of Rectal Swabs	Critical Wards				Overall
		Hematology	Intensive Care	Medicine	Surgery	
January 2024	Total number of swabs	12	30	224	49	315
	Number (%) of positive swabs	1 (8.3)	1 (3.3)	12 (5)	2 (4.1)	17 (5.4)
February 2024	Total number of swabs	27	58	379	91	555
	Number (%) of positive swabs	1 (3.7)	2 (3.4)	21 (5.5)	0 (0.0)	24 (4.3)
March 2024	Total number of swabs	22	79	426	80	607
	Number (%) of positive swabs	2 (9.1)	3 (3.7)	16 (3.7)	0 (0.0)	21 (3.5)
April 2024	Total number of swabs	27	53	371	75	526
	Number (%) of positive swabs	1 (3.7)	3 (5.7)	16 (4.3)	1 (1.3)	21 (4.0)
May 2024	Total number of swabs	36	46	412	78	572
	Number (%) of positive swabs	0 (0.0)	2 (4.3)	25 (6.0)	3 (3.8)	30 (5.2)
June 2024	Total number of swabs	17	30	198	41	286
	Number (%) of positive swabs	0 (0.0)	0 (0.0)	5 (2.5)	1 (1.3)	6 (2.1)

## Data Availability

All the data related to the manuscript are available in aggregative form. Data regarding concordance results between molecular assay and cultural assay per sample are available on reasonable request from the corresponding author.
